# A causal relationship between childhood obesity and risk of osteoarthritis: results from a two-sample Mendelian randomization analysis

**DOI:** 10.1080/07853890.2022.2085883

**Published:** 2022-06-15

**Authors:** Ziqin Cao, Yudi Wu, Qiangxiang Li, Yajia Li, Jianhuang Wu

**Affiliations:** a Department of Spine Surgery and Orthopaedics, Xiangya Hospital, Central South University, Changsha, China; b Nanchang University Queen Mary School, Nanchang, China; c Ningxia Geriatric Disease Clinical Research Center, People’s Hospital of Ningxia Hui Autonomous Region, Yinchuan, China; d National Clinical Research Center for Geriatric Disorders of Xiangya hospital, Central South University (Sub-center of Ningxia), Yinchuan, China; e Department of Hunan Institute of Geriatrics, Hunan People’s Hospital, Changsha, China; f Department of Dermatology, Xiangya Hospital, Central South University, Changsha, China; g National Clinical Research Center for Geriatric Disorders, Xiangya Hospital, Central South University, Changsha, China

**Keywords:** Childhood obesity, osteoarthritis, mendelian randomization

## Abstract

**Purpose:**

It has been found that childhood obesity (CO) may play an important role in the onset and progression of osteoarthritis (OA). Thus we conducted this mendelian randomisation analysis (MR) to evaluate the causal association between childhood obesity and osteoarthritis.

**Methods:**

Instrumental variables (IVs) were obtained from publicly available genome-wide association study datasets. The leave-one-out sensitivity test, MR Pleiotropy RESidual Sum and Outlier test (MR-PRESSO), and Cochran’s *Q* test were used to confirm the heterogeneity and pleiotropy of identified IVs, then five different models, including the inverse variance weighted model (IVW), weighted median estimator model (WME), weighted model-based method (WM), MR-Egger regression model (MER), and MR-Robust Adjusted Profile Score (MRAPS) were applied in this MR analysis.

**Results:**

After excluding all outliers identified by the MR-PRESSO test, no evident directional pleiotropy was found. Significant heterogeneity was found in the secondary MR and as a result, the multiplicative random-effect model was used. Significant causal association between CO and OA (OR 1.0075, 95% CI [1.0054, 1.0010], *p* = 8.12 × 10^−13^). The secondary MR also revealed that CO was causally associated with knee OA (OR 1.1067, 95% CI [1.0769, 1.1373], *p* = 3.30 × 10^−13^) and hip OA (OR 1.1272, 95% CI [1.0610, 1.1976], *p* = 1.07 × 10^−4^). The accuracy and robustness of these findings were confirmed by sensitivity tests.

**Conclusion:**

There appears to be a causal relationship between childhood obesity and OA. Our results indicate that individuals with a history of childhood obesity require specific clinical attention to prevent the development of knee and hip OA.

## Introduction

Osteoarthritis (OA) is a common progressive chronic degenerative joint disease, which could result in pain, disability, and increased health and socioeconomic burden. Over 250 million people worldwide are affected by OA [1,2], a disease that is characterised by pathological changes in joints such as the hands, knees, hips, or feet. While the condition is characterised by changes in articular cartilage, there is also the involvement of bone, ligament, and connective tissues. Clinically, OA manifests with progressive joint pain, stiffness, swelling, limited activity, and deformity [3,4]. Risk factors include advanced age, female sex, obesity, genetics, and major joint injury, some of which are targeted as part of preventive and therapeutic strategies.

Obesity is a global problem resulting in excessive morbidity and mortality. In recent years, considerable evidence has emerged that obesity and OA are one of the most important risk factors for peripheral joint problems, especially in the hips and knees [5–9]. In turn, weight-loss interventions have been shown to provide significant improvements in pain and disability for OA patients [10]. However, most of these studies have focussed on the association between obesity and OA in adults, and little is known about the risk of obesity and OA in childhood. Observational studies have shown that childhood obesity is associated with knee joint pain, stiffness, and dysfunction in adulthood [11,12]. However, those studies could be affected by confounders, such as health and nutritional status, which could result in a potential relationship of reverse causality. Mendelian randomisation (MR) is an analytic approach for drawing causal inferences, used in the field of epidemiological aetiology. By introducing instrumental variables as genetic predictors, the association of genes with diseases is not affected by common confounders such as the environment, socioeconomic factors, and individual behaviours [13,14]. To bridge some of the identified problems in prior studies, in this study we sought to investigate the association between childhood obesity and OA by using a two-sample MR analysis.

## Methods and materials

### Study design

Two-sample MR is considered a method of identifying the causal relationship between the phenotype of exposure and the outcome by using genetic variants for exposure as instrument variables (IV), which could make use of the accessible public dataset from large-sample genome-wide association studies (GWAS) for both “exposures” (as a risk factor) and “outcomes” (as a disease) and make up for typical shortcomings of observational studies. This study is a secondary data review of existing databases. This study was designed based on the following three assumptions: (1) the relevance assumption: that the chosen independent variables (IVs) are directly associated with the exposure of interest; (2) the independence assumption: that the chosen IVs are not associated with any confounder variables between the exposure and outcome; (3) the exclusion restriction assumption: the chosen IVs do not affect the outcome, except through their association with the exposure [15,16]. The two-sample MR analysis was used to assess the causal association of childhood obesity (exposure) with the risk of OA (the primary outcome) and its sub-type, including knee and hip OA (the secondary outcomes).

### Data source

Publicly available GWAS databases were searched to obtain eligible datasets of exposure and outcomes, including GWAS catalog, nealelab, IEU openGWAS, and PheWeb databases. As such, no additional ethical approvals were required. Considering that the confounding of the population can lead to biased estimates, we limited the genetic background of the population for the MR study to individuals of European descent.

The summary-level data on childhood obesity were obtained from a genome-wide association meta-analysis (GWAS ID: ieu-a-1096) conducted by the Early Growth Genetics (EGG) consortium [17]. In this dataset, 13,848 European children (5530 OA cases and 8318 controls) were analysed and 2,442,739 single-nucleotide polymorphisms (SNPs) were identified.

Primary outcome data were obtained from a publicly available GWAS dataset (GWAS ID: ukb-b-14486). This dataset was built by the MRC Integrative Epidemiology Unit (MRC-IEU) consortium using the UK Biobank and contained 462,933 Europeans (38,472 cases and 424,461 controls) with 9,851,867 SNPs. The two datasets (GCST007090 and GCST007091) used in the secondary outcomes were obtained from the same GWAS study by Tachmazidou et al. [18]. The dataset GCST007090 included 403,124 Europeans (38,472 knee osteoarthritis patients and 424,461 healthy controls) and 29,999,696 SNPs, while the dataset GCST007091 included 393,873 Europeans (15,704 knee osteoarthritis patients and 378,169 healthy controls) and 29,771,219 SNPs.

### SNP in exposure and outcome selection

The GWAS database was searched for SNPs selection according to the above assumptions. All SNPs would be clumped to avoid the linkage disequilibrium under a strict clump window (*r*^2^ = 0.001 and *kb* = 10,000). When the threshold was set as *p* < 5 × 10^−8^, only six SNPs could be identified thus failing to meet the minimum requirements for MR studies of at least 10 eligible IVs [19,20]. As such, 15 SNPs were selected using a less stringent threshold of *p* < 5 × 10^−6^ [21,22] and were detected at phenome-wide association studies (pheWAS) catalog databases to identify whether there was a potential association of these SNPs with confounders of outcomes, with a threshold of *p* < 5 × 10^−6^ [22,23]. *F* statistics were calculated to estimate the sample overlap effect and weak instrument bias considering the relatively relaxed threshold, and an *F* < 10 was considered dubious bias [24]. The SNP rs1040070 was further removed as it was palindromic with intermediate allele frequencies. The details of 14 finally identified IVs are presented in Table 1. Summary statistics of childhood obesity and OA have harmonised in terms of effect allele, and subsequent analyses were based on the merged exposure-outcome dataset.

**Table 1.** The detailed information of identified SNPs in exposure and outcomes.

SNP					Exposure (Childhood Obesity)				Primary Outcome (Osteoarthritis)				Secondary Outcome (Knee Osteoarthritis)				Secondary Outcome (Hip Osteoarthritis)				*F*
RS ID	Chromosome	Position	EA	OA	Beta	Se	*p*-Value	EAF	Beta	Se	*p*-Value	EAF	Beta	Se	*p*-Value	EAF	Beta	Se	*p*-Value	EAF	
rs10913469	1	177913519	C	T	0.1773	0.0330	7.99E-08	NA	0.0016	0.0007	2.10E-02	0.204573	0.0364	0.0115	1.53E-03	0.2061	0.0325	0.0144	2.42E-02	0.206	14.95681884
rs13130484	4	45175691	T	C	0.1434	0.0272	1.30E-07	NA	0.0011	0.0006	5.90E-02	0.433632	0.0124	0.0094	1.87E-01	0.4344	0.0072	0.0118	5.40E-01	0.435	22.24119754
rs17697518	18	38765659	T	C	0.1855	0.0389	1.85E-06	NA	0.0004	0.0009	6.00E-01	0.127075	0.018	0.0139	1.95E-01	0.1277	−0.0103	0.0175	5.55E-01	0.1274	8.982757484
rs256335	19	34315896	T	C	0.1214	0.0262	3.72E-06	NA	0.0014	0.0006	1.80E-02	0.468476	0.0066	0.0093	4.81E-01	0.4681	0.0057	0.0117	6.24E-01	0.4682	19.76190384
rs28636	5	66149113	T	C	−0.1474	0.0316	3.07E-06	NA	−0.0010	0.0007	1.50E-01	0.22	−0.0061	0.0112	5.84E-01	0.2208	−0.0107	0.0141	4.47E-01	0.2209	13.65253784
rs4833407	4	113311790	A	C	0.1226	0.0265	3.88E-06	NA	0.0005	0.0006	3.60E-01	0.403415	0.0096	0.0095	3.08E-01	0.4029	0.0034	0.0119	7.73E-01	0.4038	19.05791264
rs4854344	2	638144	T	G	0.2445	0.0351	3.22E-12	NA	0.0026	0.0008	5.20E-04	0.827819	0.023	0.0123	6.17E-02	0.8287	0.0268	0.0155	8.26E-02	0.829	16.90468749
rs4864201	4	130731284	C	T	−0.1355	0.0281	1.41E-06	NA	−0.0016	0.0006	6.40E-03	0.650954	−0.0197	0.0097	4.26E-02	0.6534	0.0063	0.0122	6.06E-01	0.6539	18.70192717
rs571312	18	57839769	A	C	0.1986	0.0309	1.25E-10	NA	0.0018	0.0007	9.20E-03	0.233489	0.0305	0.011	5.38E-03	0.2334	0.0503	0.0137	2.50E-04	0.2342	19.71414151
rs6752378	2	25150116	A	C	0.1695	0.0262	1.05E-10	NA	0.0014	0.0006	1.80E-02	0.486255	−0.0221	0.0093	1.75E-02	0.4869	0.0162	0.0117	1.64E-01	0.4879	27.90849147
rs7138803	12	50247468	A	G	0.1672	0.0271	6.50E-10	NA	0.0013	0.0006	2.60E-02	0.369175	0.0111	0.0096	2.50E-01	0.3683	0.0505	0.0121	2.85E-05	0.3686	24.71880986
rs9299	17	46669430	T	C	0.1343	0.0282	1.91E-06	NA	0.0005	0.0006	4.10E-01	0.656465	0.0132	0.0098	1.75E-01	0.6574	0.0331	0.0123	6.96E-03	0.6579	18.3504962
rs9568856	13	54064981	A	G	0.1909	0.0395	1.36E-06	NA	0.0003	0.0009	7.30E-01	0.128121	0.0095	0.0139	4.95E-01	0.1276	0.0121	0.0174	4.85E-01	0.128	9.136671867
rs9941349	16	53825488	T	C	0.1978	0.0267	1.16E-13	NA	0.0011	0.0006	5.40E-02	0.41273	0.044	0.0094	3.00E-06	0.4123	0.0499	0.0118	2.44E-05	0.4126	31.09879584

EA: effect allele; OA: other allele; EAF: NA: not applicable.

### Statistical analysis

This two-sample MR analysis was performed using R software (version 4.1.2, R Foundation for Statistical Computing, Vienna, Austria) with TwoSampleMR (version 0.5.6) and MR-PRESSO packages (version 1.0.0).

The classic inverse variance weighted model (IVW) was employed in the primary MR analyses. When directional pleiotropy is absent, the IVW method can deliver a relatively stable and accurate causal evaluation by using a meta-analytic approach to combine Wald estimates for each IV [25,26].

The weighted median estimator model (WME), weighted model-based method (WM), MR-Egger regression model (MER), and MR-Robust Adjusted Profile Score (MRAPS) were also used to estimate causal effects. The WME can obtain a robust result when more than 50% of weights came from invalid IVs and reduce the type I error to evaluate a more accurate causal association if horizontal pleiotropy exists [27], while the WM method can obtain a robust overall causal estimate when the majority of similar individual estimates were from valid IVs [28]. The MER method can provide a relatively robust estimate without the influence of the validity of IVs, and an adjusted result by existing horizontal pleiotropy *via* the regression slope and intercept [29,30]. However, compared to the IVW method, the WME, ME, and MER methods have compromised power, as indicated by wide confidence intervals (CI) [31], and would only serve as complementary methods in this study. MRAS could obtain a more accurate causal assessment if the independence of IVs is perfected [32], and hence also would serve as a complementary method.

The heterogeneity between IVs was tested by Cochrane’s *Q*-statistic. Significant heterogeneity was indicated if *p* < .05, and a random-effect model would be adopted in the subsequent analyses, otherwise, a fixed-effect model would be adopted [33]. The leave-one-out sensitivity test was used to judge the stability of the MR results by excluding IVs one by one [34]. Directional pleiotropy was checked and corrected based on the intercept obtained from the MER analysis [30] and the MR pleiotropy residual sum and outlier test (MR-PRESSO). In addition, the effects of outlying IVs identified by MR-PRESSO tests were evaluated in a further distortion test, and any outliers whose *p* < .05 in the distortion test would be excluded and the causal estimates would be reassessed [35]. Causal estimates were given as odds ratios (ORs) and 95% confidence intervals. An adjusted *p*-value of .01 after Bonferroni correction (*p* < .05/*N*, *N* = testing methods number) was considered statistically significant.

## Results

### Primary MR analysis of childhood obesity and osteoarthritis

There was no evidence of heterogeneity (*Q* = 7.696089, *p* = .8628) in the Cochran’s *Q* test, and hence a fixed-effects model was adopted in the primary MR analysis. The IVW analysis found a significant causal association between childhood obesity and OA (OR 1.0075, 95% CI [1.0054, 1.0010], *p* = 8.12 × 10^−13^), and was replicated *via* WME (OR 1.0079, 95% CI [1.0050, 1.0107], *p* = 5.53 × 10^−8^), WM (OR 1.0081, 95% CI [1.0037, 1.0125], *p* = 2.98 × 10^−3^), and MRAPS (OR 1.0078, 95% CI [1.0056, 1.0101], *p* = 6.88 × 10^−12^) analyses. No outlier between childhood obesity and the risk of osteoarthritis was identified by the MR-PRESSO test, and the robustness of results was confirmed by the leave-one-out sensitivity test. No directional pleiotropy was found in the MR-Egger regression (intercept = −0.0001, se = 0.0009, *p* = .8995) and MR-PRESSO Global test (Rssobs = 8.8444, *p* = .9080).

### Secondary MR analysis of childhood obesity and knee osteoarthritis

Significant heterogeneity (*Q* = 30.0517, *p* = .0046) was found *via* the Cochran’s *Q* test, and hence a multiplicative random-effect model was adopted in this MR analysis. The MR-PRESSO global test reported two outliers (rs6752378, Rssobs = 0.002, *p* < .0140; and rs9941349, Rssobs = 0.0008, *p* = .0420), and a significant distortion was detected. After removing these two outliers, a significant causal association was observed in the IVW analysis between childhood obesity and knee OA (OR 1.1067, 95% CI [1.0769, 1.1373], *p* = 3.30 × 10^−13^), and was replicated *via* WME (OR 1.0952, 95% CI [1.0394, 1.1541], *p* = 6.58 × 10^−4^) and MRAPS (OR 1.1103, 95% CI [1.0686, 1.1536], *p* = 8.46 × 10^−8^) analyses. WM analysis showed a contributory but not significant effect of childhood obesity on the risk of knee OA (OR 1.0839, 95% CI [1.0070, 1.1669], *p* = 5.51 × 10^−2^). The robustness of results was confirmed by the leave-one-out sensitivity test. No directional pleiotropy was found in the MR-Egger regression (intercept = −0.0164, se = 0.0218, *p* = .4659) and MR-PRESSO Global test (Rssobs = 7.0378, *p* = .9110).

### Secondary MR analysis of childhood obesity and hip osteoarthritis

The Cochran’s *Q* test identified heterogeneity across the included IVs (*Q* = 26.75145, *p* = .0135), and a multiplicative random-effect model was used in this MR analysis. The IV rs9941349 (Rssobs = 0.0009, *p* = .0470) was identified as an outlier in the MR-PRESSO test and was excluded from the subsequent analyses. The IVW analysis reported a significant causal relationship between childhood obesity and hip OA (OR 1.1272, 95% CI [1.0610, 1.1976], *p* = 1.07 × 10^−4^), and was consistent with the findings of the WME (OR 1.0944, 95% CI [1.0216, 1.1723], *p* = 1.02 × 10^−2^) and MRAPS analyses (OR 1.1238, 95% CI [1.0536, 1.1987], *p* = 3.89 × 10^−4^). The robustness of results was confirmed by the leave-one-out sensitivity test. No directional pleiotropy was found in the MR-Egger regression (intercept = −0.0229, se = 0.0254, *p* = .3845) and MR-PRESSO Global test (Rssobs = 26.5238, *p* = .057).

**Figure 1.**
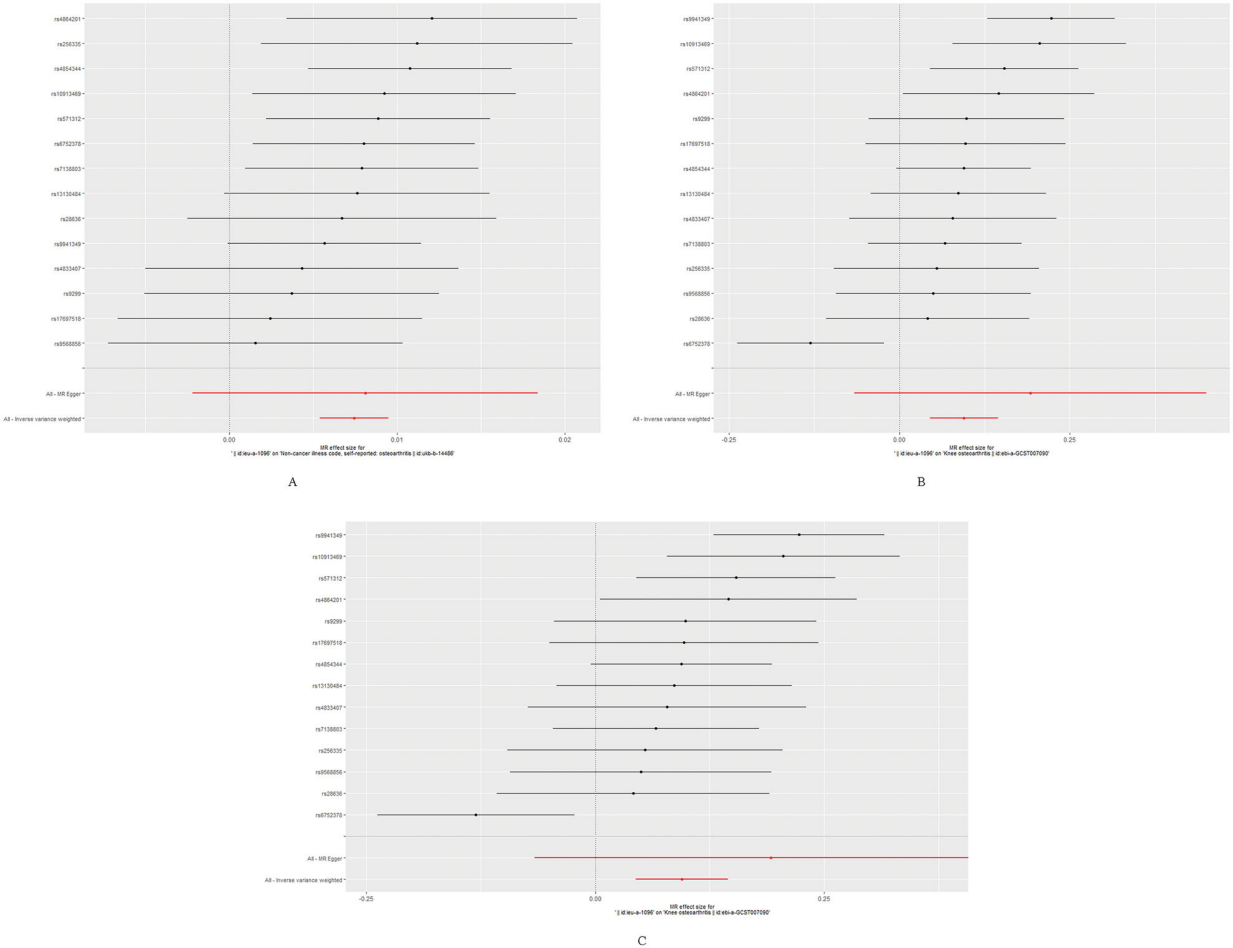
Detailed forest plots with the estimated MR effect of each IV in IVW models. (A) Primary outcome (Osteoarthritis); (B) Secondary outcome (Knee Osteoarthritis); (C) Secondary outcome (Hip Osteoarthritis).

**Figure 2.**
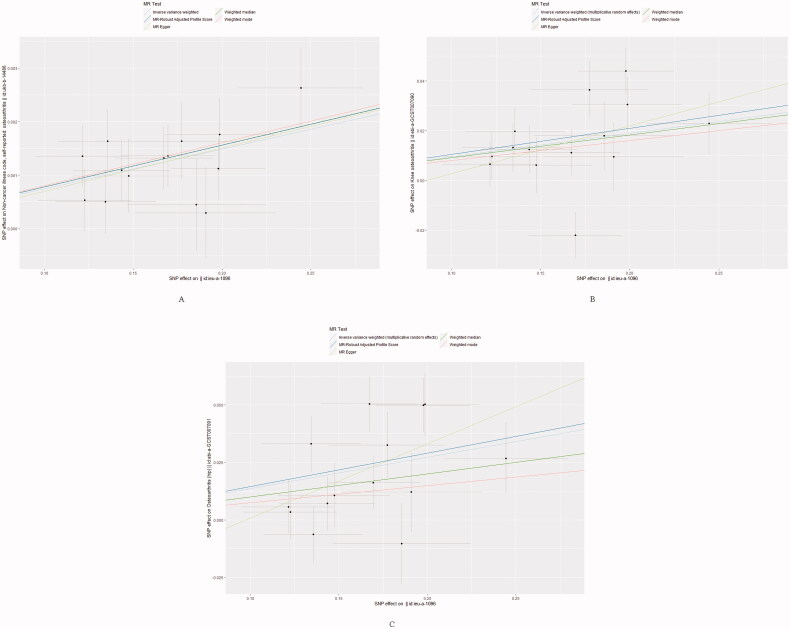
Scatter plots of causality. The slope of each line corresponding to the estimated MR effect in different models. (A) Primary outcome (Osteoarthritis); (B) Secondary outcome (Knee Osteoarthritis); (C) Secondary outcome (Hip Osteoarthritis).

**Figure 3.**
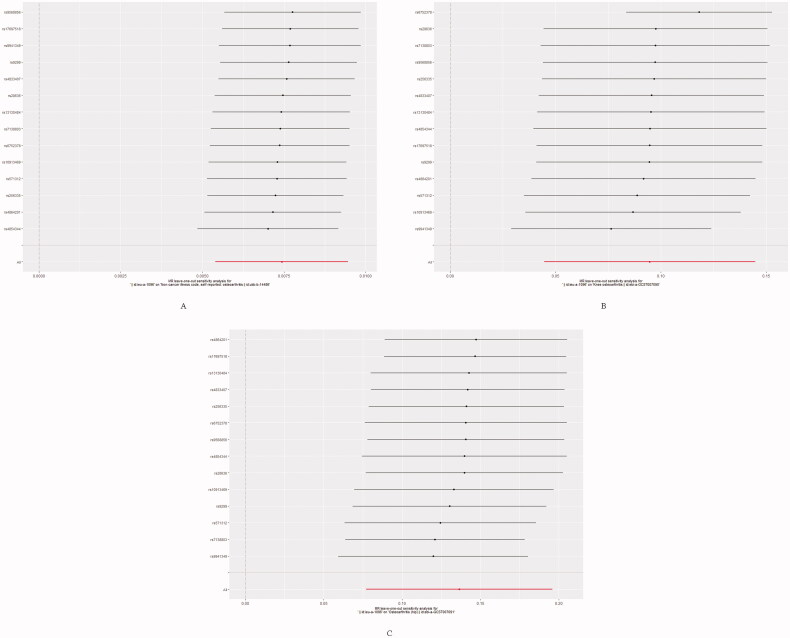
Leave one out of sensitivity tests. Calculate the MR results of the remaining IVs after removing the IVs one by one. (A) Primary outcome (Osteoarthritis); (B) Secondary outcome (Knee Osteoarthritis); (C) Secondary outcome (Hip Osteoarthritis).

**Figure 4.**
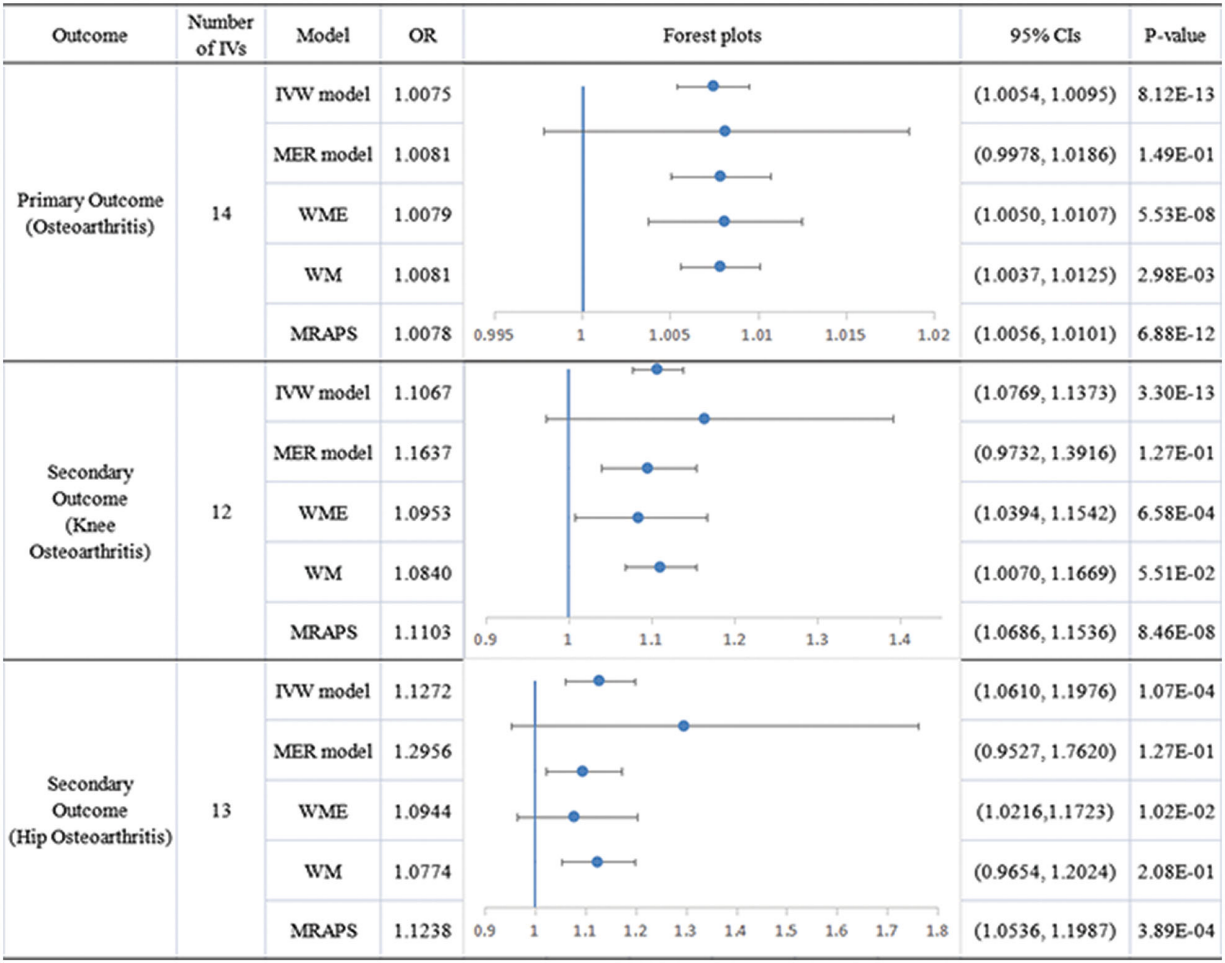
Causal estimates given as odds ratios (ORs) and 95% confidence intervals for the effect of childhood obesity on osteoarthritis and its sub-types.

The forest plot and scatter plot of causal relationships between genetically predicted childhood obesity and the risk of osteoarthritis and its subtypes are shown in Figures 1 and 2, and the details of sensitivity analyses are shown in Figures 3 and Table 2. Detailed causal effect estimates for associations between exposure and outcomes in different models were presented in Figure 4.

**Table 2.** Sensitivity analysis of primary and secondary MR analyses.

Outcome	Number of IVs	Heterogeneity test		MR-Egger pleiotropy test		MR-PRESSO global pleiotropy test		
		Q	*p*-Value	Intercept	*p*-Value	RSSobs	*p*-Value	Outliers
Primary Outcome (Osteoarthritis)	14	7.6961	.8628	−0.000111772	.8995	8.844354	.908	None
Secondary Outcome (Knee Osteoarthritis)	12	30.0517	.0046	−0.01637855	.4660	7.037842	.911	rs6752378, rs9941349
Secondary Outcome (Hip Osteoarthritis)	13	26.7515	.0135	−0.03142383	.2287	26.52378	.057	rs9941349

IVs: instrumental variables.

## Discussion

Our MR study revealed a causal relationship between CO and OA. A significant causal association between genetic risk of CO and OA with an OR of 1.0075, especially for knee OA (OR 1.1067, 95% CI [1.0769, 1.1373]) and hip OA (OR 1.1272, 95% CI [1.0610, 1.1976]). Previous observational studies and reviews have reported an association between obesity and OA. Salis et al. [36] conducted a time-to-event survival analysis, using a population-based cohort with a high risk of clinically significant knee OA to determine the association between body weight change and the risk of subsequent knee and/or hip replacement. 8145 individuals were included (8069 knees and 8076 hips). They reported that every 1% reduction in weight was associated with an almost 2% (knee)–3% (hip) reduction in the risk of joint replacement, suggesting that obesity promotes the development of OA. A study by Wills et al. [37] analysed a British cohort of 3035 individuals and reported that changes in early-life BMI were positively associated with knee OA in men and women. A 25-year longitudinal cohort study [12] composed of 449 Australians (aged 31–41 years, female 48%) utilised a comprehensive assessment of weight, height, and knee symptoms (Western Ontario MacMaster Universities osteoarthritis index [WOMAC]). They similarly reported that childhood overweight was significantly associated with later knee symptoms, including pain (RR 1.68, 95% CI [1.06–2.65]), stiffness (RR 1.10, 95% CI [1.02–1.18]), and dysfunction (RR 1.52, 95% CI [0.99–2.32]) in men, and was independent of the adult overweight status. These data suggest that childhood obesity may be an independent risk factor for knee OA. While significant efforts have been made to improve the treatment and prevention of OA, its contributory mechanisms are still incompletely understood. There multiple additional studies have focussed on the mechanisms linking childhood obesity and an increased risk of OA. Molina-Garcia et al. [38]. investigated the relationship between obesity and altered knee joint biomechanics in children. Their study provided moderately strong evidence that childhood obesity is associated with a compensatory gait which, while maintaining a normal knee extensor load, can lead to increased medial compartment joint loads. The systematic review by Molina-Garcia et al. [39]. also found significant biomechanical differences in the gait patterns of overweight and obese children and adolescents, including a greater range of pelvic, hip, knee, and ankle plantar motion, and a higher torque and power generation/absorption. These findings indicate that the biomechanical abnormalities observed in childhood obesity may contribute to the onset and progression of musculoskeletal disorders such as OA.

Our MR study is the first to comprehensively evaluate the causal link between childhood obesity and OA. We identified 14 SNPs using three GWAS datasets and using five different models to identify the causal relationship. In our analyses, we found directional pleiotropy and adjusted for it by applying the MR-PRESSO test after excluding all dubious outliers. Therefore, the results from the IVW and IVW multiplicative random-effect models were selected. A robust causal association of childhood obesity with OA, including knee and hip osteoarthritis, was observed in our study. The sensitivity test supported the stability and accuracy of the causal outcome additionally. The results of our study provided the evidence that genetic risk of childhood obesity was directly associated with osteoarthritis, and early prevention and clinical intervention for OA diseases could be taken into consideration for a population with childhood obesity.

There are some limitations to our work. First, few SNPs fell under the standard bioinformatic threshold of *p* < 5 × 10^−8^. This number of SNPs would make it difficult to match IVs in the outcome, but could also weaken any associations. As such, we selected SNPs using a less stringent significance of 5 × 10^−6^. This approach has been suggested in previous studies [21,22], with the limitation that it can cause weak instrumental variable bias. We calculated F statistics to assess the risk of such bias and did not find strong evidence of its existence (except for rs9568856 [*F* = 9.1367] and rs17697518 [*F* = 8.9828], other IVs all showed an *F* value greater than 10). However, we still suggest some caution in interpreting our results. Second, the only publicly available GWAS of childhood obesity does not report the specific features of childhood obesity such as weight, height, and abdominal circumference. As such, it is impossible to further classify childhood obesity and to conduct a stratified MR analysis based on the obesity class. This would be helpful to draw more accurate causal inferences with more control over potential confounders. Besides that, we limited the genetic background of the population for the MR study to individuals of European ancestry to avoid potential confounding from a more heterogeneous population. However, we acknowledge that this limits the confidence with which we can extrapolate from our results to those of different races. There appears to be horizontal pleiotropy if the second phenotype presents on a different biological pathway, thus, different causal pathways may exist for the variant to the outcome, which could violate the assumption of IV3. In dealing with the horizontal pleiotropy, some robust MR methods except for IVW were also used in this study, and different methods had their advantages. Moreover, Selected SNPs were also matched to pheWAS databases for avoiding the confounders, and associated horizontal pleiotropy with a threshold of *p* < 5 × 10^−6^. But these measures could not avoid the horizontal pleiotropy effect completely because it was difficult to fully discovered the exact biological function of many genetic variants. More high-quality GWAS and MR analyses are thus needed in the future.

## Conclusion

In conclusion, there appears to be a causal relationship between childhood obesity and OA. Our results indicate that individuals with a history of childhood obesity require specific clinical attention to prevent the development of knee and hip OA. Further studies are needed to examine the biological mechanisms underlying this association.

## Data Availability

The data that support the findings of this study are available from the corresponding author upon reasonable request.

## Abbreviations

CO: childhood obesity
EGG: the Early Growth Genetics consortium
GWAS: genome-wide association studies
IVs: Instrumental variables
IVW: inverse variance weighted model
MER: MR-Egger regression model
MR: Mendelian randomisation analysis
MR-PRESSO: MR Pleiotropy RESidual Sum and Outlier test
MRAPS: MR-Robust Adjusted Profile Score
MRC-IEU: the MRC Integrative Epidemiology Unit consortium
OA: osteoarthritis
pheWAS: phenome-wide association studies catalog databases
SNPs: single-nucleotide polymorphisms
WM: weighted model-based method
WME: weighted median estimator model
WOMAC: Western Ontario MacMaster Universities osteoarthritis index.
